# Fetal heart rate development during labour

**DOI:** 10.1186/s12938-021-00861-z

**Published:** 2021-03-16

**Authors:** Jarle Urdal, Kjersti Engan, Trygve Eftestøl, Solveig H. Haaland, Benjamin Kamala, Paschal Mdoe, Hussein Kidanto, Hege Ersdal

**Affiliations:** 1 Department of Electrical Engineering and Computer Science, University of Stavanger, Stavanger, Norway; 2 Strategic Research, Laerdal Medical AS, Stavanger, Norway; 3 Haydom Lutheran Hospital, Haydom, Manyara Tanzania; 4 School of Medicine, Aga Khan University, Dar es Salaam, Tanzania; 5 Department of Anesthesiology and Intensive Care, Stavanger University Hospital, Stavanger, Norway; 6 Faculty of Health Sciences, University of Stavanger, Stavanger, Norway; 7 Department of Obstetrics and Gynecology, Muhimbili National Hospital, Dar es Salaam, Tanzania

**Keywords:** Fetal heart rate, Perinatal mortality, Signal processing

## Abstract

**Background:**

Fresh stillbirths (FSB) and very early neonatal deaths (VEND) are important global challenges with 2.6 million deaths annually. The vast majority of these deaths occur in low- and low-middle income countries. Assessment of the fetal well-being during pregnancy, labour, and birth is normally conducted by monitoring the fetal heart rate (FHR). The heart rate of newborns is reported to increase shortly after birth, but a corresponding trend in how FHR changes just before birth for normal and adverse outcomes has not been studied. In this work, we utilise FHR measurements collected from 3711 labours from a low and low-middle income country to study how the FHR changes towards the end of the labour. The FHR development is also studied in groups defined by the neonatal well-being 24 h after birth.

**Methods:**

A signal pre-processing method was applied to identify and remove time periods in the FHR signal where the signal is less trustworthy. We suggest an analysis framework to study the FHR development using the median FHR of all measured heart rates within a 10-min window. The FHR trend is found for labours with a normal outcome, neonates still admitted for observation and perinatal mortality, i.e. FSB and VEND. Finally, we study how the spread of the FHR changes over time during labour.

**Results:**

When studying all labours, there is a drop in median FHR from 134 beats per minute (bpm) to 119 bpm the last 150 min before birth. The change in FHR was significant ($p<0.05$) using Wilcoxon signed-rank test. A drop in median FHR as well as an increased spread in FHR is observed for all defined outcome groups in the same interval.

**Conclusion:**

A significant drop in FHR the last 150 min before birth is seen for all neonates with a normal outcome or still admitted to the NCU at 24 h after birth. The observed earlier and larger drop in the perinatal mortality group may indicate that they struggle to endure the physical strain of labour, and that an earlier intervention could potentially save lives. Due to the low amount of data in the perinatal mortality group, a larger dataset is required to validate the drop for this group.

## Background

Fetal heart rate (FHR) monitoring is a widely used method to assess the status of the fetus during pregnancy, labour and birth. In high-resource countries, continuous monitoring of the FHR is done using cardiotocography (CTG) for labours categorised as high risk. In low-income and low-middle income countries (LMIC), an intermittent measurement is the norm for all labours. The intermittent measurement is normally conducted using a Pinard stethoscope or hand-held Doppler. Guidelines state that auscultation of FHR should be conducted every 15–30 min during the first stage of labour, and every 5–15 min during the second stage of labour. Each auscultation should also last for at least 1 min [1]. The intervals defined by the guidelines is not possible without a nurse:patient ratio of 1:1 [2] and will be a challenge in LMIC where the ratio of health care workers to the number of labours is much lower. A limitation of intermittent auscultation, independent of the device used, is that the status of the fetus is only checked during a specific point in time. When the time between each auscultation increase, the possibility of detecting an abnormal FHR may be reduced.

Stillbirths is a worldwide challenge, with an estimated 2.6 million [uncertainty range 2.4–3.0] stillbirths in 2015 [3], of these 1.3 million is estimated to have died during labour and birth, i.e. fresh stillbirth (FSB). In addition, 1 million newborns die within their first and only day of life [3, 4]. Asphyxia and prematurity complications during labour are the primary causes of death. The vast majority, 98%, of stillbirth and early neonatal death are found in LMIC settings [3]. The use of continuous FHR monitoring devices in LMIC settings may help the health care workers detect abnormalities in FHR at an earlier stage, allowing the health care personnel time to intervene before it is too late.

The heart rate of newborns the first 5 min and the first 24 h after birth have previously been studied [5, 6]. It was shown that the normal HR after birth increase significantly in spontaneously breathing infants the first minutes after birth from 120 to 160 bpm in median. These numbers suggest the hypothesis that the fetal HR goes down right before birth, in the very last phase of the labour. Current guidelines states that FHR in the range 110–160 bpm during labour is considered normal [7, 8], but a study of how the FHR develops at the last part of the labour has, to the authors knowledge, never been presented before.

Extensive work has been applied by different research groups to analyse continuous FHR measurements [9–12]. Most of the effort has been on interpreting FHR according to existing guidelines, mimicking the way an experienced health care provider would interpret the CTG. Lately, the focus has shifted more towards the use of deep learning for these analyses [13, 14]. More research is needed to establish how different FHR patterns are connected to outcomes like stillbirth, early neonatal death, and/or need for resuscitation after birth.

This work is a part of the larger Safer Birth project 1, a collaboration between multiple Norwegian and international research institutions as well as hospitals in Tanzania. The aim of Safer Births is to increase newborn survival by gaining new knowledge and developing innovative products to aid the help care workers. Among the data that has been collected through the Safer Births project, FHR from 3711 labours in Tanzania was collected using the Moyo fetal heart rate monitor 2, a Doppler-based ultrasound sensor developed for continuous or intermittent FHR monitoring, useful also in LMIC settings.

The main objectives of this paper are to present a framework that can be used to analyse fetal heart rate collected continuously using Moyo (or potentially other FHR devices). The framework will be used on the Safer Births data to study: (1) the maternal-fetal heart rate ambiguity for the Moyo device, and compare that to reported ambiguities using a traditional CTG. (2) The development of the FHR the last part of the labour on labours assessed as normal at admission. (3) The fetal heart rate probability density functions for the last part of the labour divided into groups based on the neonatal outcome 24 h after birth.

Understanding how the FHR develops differently for the different neonatal outcome groups might provide valuable insights in understanding the risk of birth asphyxia at an early phase in the labour. Automated interpretation of FHR combined with such knowledge can provide improved tools on risk prediction and decision support during labour.

## Results

**Fig. 1:**
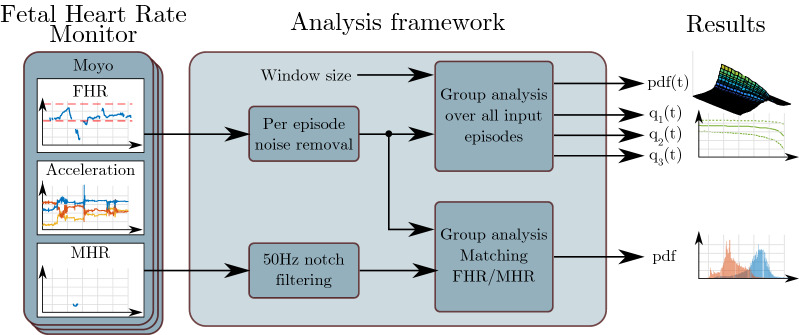
Signals acquired from the Moyo fetal heart rate monitor is used as the input for our analysis framework in this work. Noise is first removed on the FHR signals, before group analysis is conducted. The acceleration signal present in the Moyo has previously been shown to identify contractions [15], but it is not used in the work presented in this paper

The proposed FHR analysis framework is shown in Fig. 1. Each episode is first processed to remove noise, before group analyses over all inputs are conducted. Both MHR/FHR (maternal heart rate/fetal heart rate) ambiguity and FHR development for different neonatal outcome groups are analysed. The technical details of the experiments are described in "Methods" section.

The dataset were divided into groups based on the newborn outcome 24 h after birth. These outcomes were *Normal*, $s_1$, *still admitted to neonatal care unit (NCU)*, $s_2$, *very early neonatal death (VEND)*, $s_4$, and *FSB*, $s_5$. An overview of the groups are shown in Table 1. Due to the low number of episodes in $s_4$ and $s_5$, these groups were combine to describe all episodes ending with death, $s_3$, known as perinatal mortality. A total of six episodes were not labelled using the four groups above and were, therefore, excluded. The subsequent experiments on development of FHR trend were conducted using the cleaned version of the FHR signals.

**Table 1:** Overview of dataset and the removed noise from FHR signal in each labour outcome group

Outcome	#Episodes	Group	Missing data (%)	Removed data (%)
All	3711			
All labeled data	3705	$s_0$		
Normal	3490	$s_1$	$27.83\pm 19.87$	$1.79\pm 1.35$
NCU	185	$s_2$	$31.28\pm 20.38$	$1.82\pm 1.31$
VEND	18	$s_4$	$29.22\pm 24.34$	$1.31\pm 0.83$
FSB	12	$s_5$	$40.50\pm 28.60$	$1.92\pm 1.61$
Perinatal mortality	30	$s_3 = s_4 \cup s_5$		
Unlabeled data	6			

### Noise removal

**Fig. 2:**
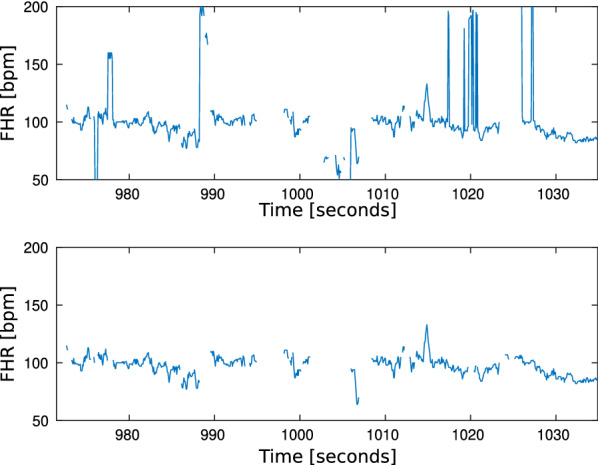
Example of the result using FhrClean. The original FHR signal with noise on top, and the corresponding signal with noise removed, bottom

**Fig. 3:**
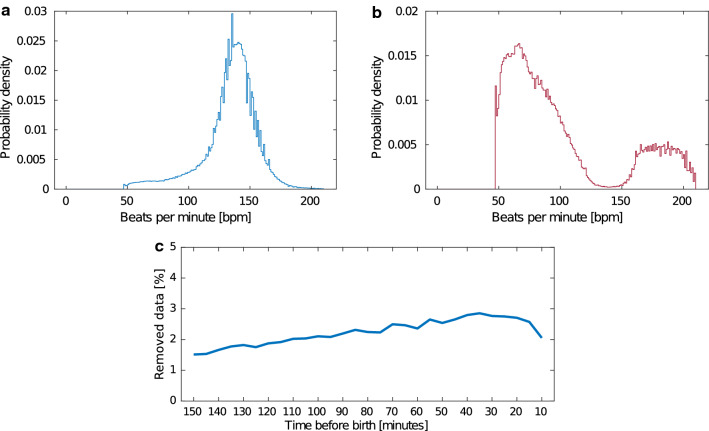
Overview of the probability density for all measured heart rates in **a**, the probability density for the removed heart rates in **b**, and the percentage of heart rate measurements identified as noise and removed the last 150 min before birth in **c**

The noise removal algorithm, FhrClean [15], was run on the entire dataset. As no truth data are available describing which Doppler measurements contain true FHR measurements or noise, FhrClean only removes segments of $<30 \, \text{s}$ with changes that are physiological impossible, as we proposed in FhrClean [15]. An example of the original and cleaned FHR signal is shown in Fig. 2 and an overview of the probability density for all measured heart rates as well as the probability density for removed heart rates in Fig. 3. While the density function for all heart rates, Fig. 3a, has its peak in the 140 bpm region, we see that most of the removed measurements, Fig. 3b, are either below 120 bpm or above 160 bpm. The percentage of removed measurements during the last 150 min before birth is computed using 5-min intervals and is shown in Fig. 3c.

An overview of the mean and standard deviation of missing data and the percentage of removed sample points for each of the four outcome groups are shown in Table 1.

### Experiment 1: heart rate ambiguity

The MHR (maternal heart rate) and FHR measurements are extracted from all episodes in the dataset. The dry-electrode ECG sensor for MHR is used in 30.54% of the episodes in the dataset. In these episodes, the MHR is measured in $0.412\pm 0.542$% of the episode duration, resulting in a total amount of 32 h where both recordings are available.

**Fig. 4:**
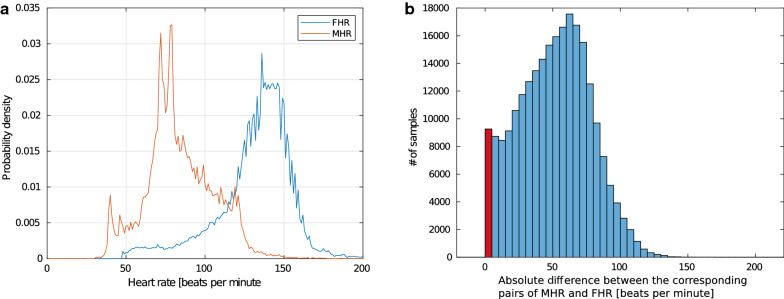
**a** Distribution of the MHR and FHR from all time points in the dataset where both values are measured. **b** The absolute difference between the corresponding pairs of MHR and FHR. The found MHR/FHR ambiguity of 4.53% is illustrated with the red bar

The distribution of the measured FHR and MHR in all time points where both values are measured are shown in Fig. 4a. The red indicates measured MHR, and blue indicates the measured FHR. The absolute difference between the measured FHR and FHR at each time point is shown in Fig. 4b. The MHR/FHR ambiguity refers to timepoints where it is not possible to distinguish measured MHR from FHR, defined in section "Heart rate ambiguity" in section "Methods". The MHR/FHR ambiguity in time points where both heart rates are measured is 4.53%, illustrated by the red bar in Fig. 4b. The similarity threshold, $T_{\text{mhr}}$, is set to 5 when computing the ambiguity, according to the study of Reinhard et al. [16].

### Experiment 2: fetal heart rate development

The FHR development is found as the median FHR, denoted $\text{mFHR}(t)$ in the following, as a function of time using non-overlapping intervals of fixed size. More details of $\text{mFHR}(t)$ can be found in the section "Fetal heart rate development" in the section "Methods". The defined starting point is set to −9000 s, equivalent of 150 min before birth.

Intervals of 5- and 10-min duration were used to obtain multiple resolutions of the heart rate trend in the period from 150 min before birth until the time of birth.

**Fig. 5:**
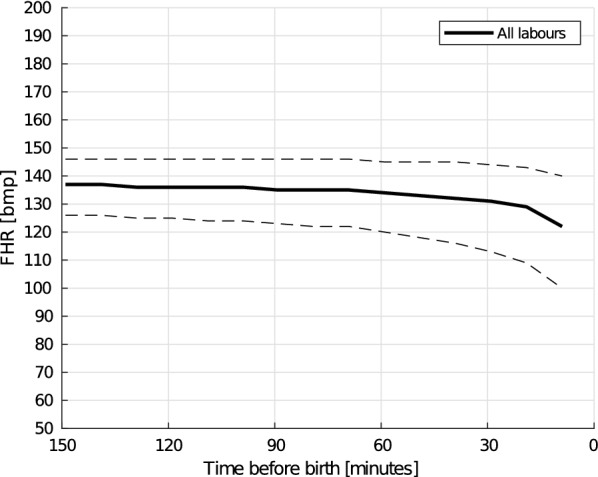
Trend of the FHR the last 150 min before birth. Solid lines indicate the median heart rate, and the matching dashed lines the 25 and 75 percentiles. Computed using non-overlapping 10-min intervals

An overview of the heart rate development for all labours using a 10-min interval is shown in Fig. 5. The solid line shows the $\text{mFHR}(t)$, and dashed lines the 1st (25%) quartile and the 3rd (75%) quartile, denoted $\text{HRq}_1(t)$ and $\text{HRq}_3(t)$ for the rest of the paper. The shift in $\text{mFHR}(t)$ from the window 150–140 min to the last 10 min before birth is found to be significant using Wilcoxon signed-rank test with $p<0.05$.

**Fig. 6:**
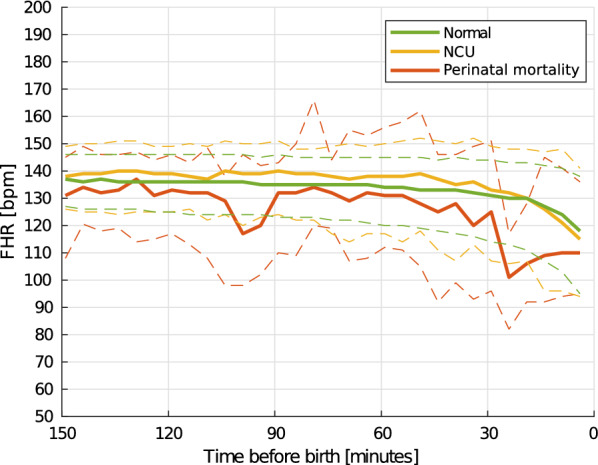
Trend of the FHR the last 150 min before birth. Solid lines indicate the median heart rate, and the matching dashed lines the 25 and 75 percentiles. Computed using non-overlapping 5-min intervals

**Fig. 7:**
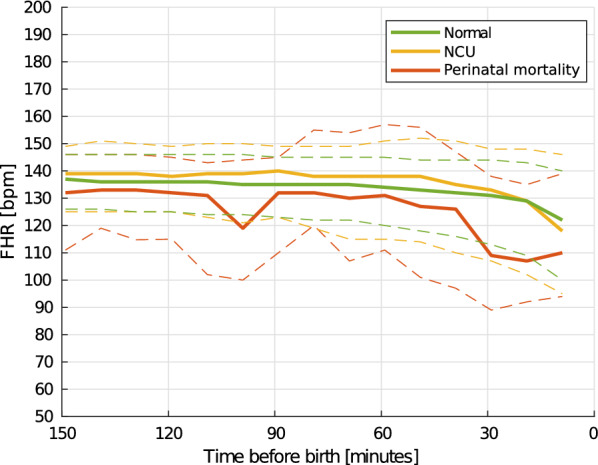
Trend of the FHR the last 150 min before birth. Solid lines indicate the median heart rate, and the matching dashed lines the 25 and 75 percentiles. Computed using non-overlapping 10-min intervals

As the FHR development during labour may also be depended on the newborns well-being, similar development curves are computed based on the newborn outcome at 24 h after birth. An overview of $\text{mFHR}(t)$ using 5- and 10-min intervals are shown in Figs. 6 and 7, respectively. The green lines shows the trend for the $s_1$ group, yellow shows for the $s_2$ group, and red line shows the $s_3$ group, perinatal mortality. The shift in $\text{mFHR}(t)$ from the window 150–140 min to the last 10 min before birth is found to be significant for both the normal and NCU group using Wilcoxon signed-rank test with $p<0.05$. Due to insufficient amount of data in the perinatal mortality group, the Wilcoxon signed-rank test assumes a normal distribution of FHR and detects a shift with $p = 0.065$.

The found trend indicates a reduction in $\text{mFHR}(t)$ the last 30 min before birth, with the reduction for the perinatal mortality group, $s_3$, occurring longer time before birth compared to for $s_1$ and $s_2$. Using the Kruskal–Wallis statistical test, the distribution of the normal and perinatal mortality group were found to be different at 20–10 min before birth with a *p*=0.064. Further validation on more data is required for the perinatal mortality group.

### Experiment 3: fetal heart rate distribution

**Fig. 8:**
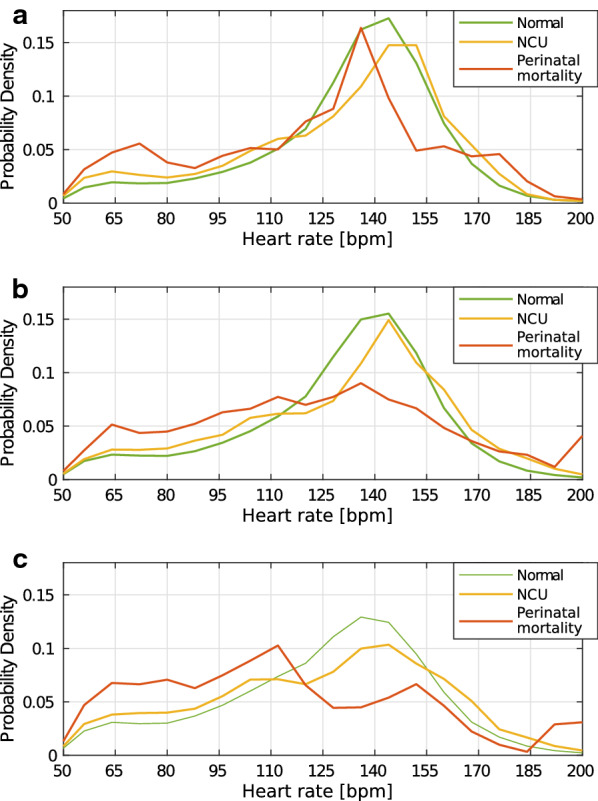
Estimated pdf of the measured heart rate the last 90 min before birth, divided in 30-min windows. **a** Estimated pdf for the last 90–60 min before birth. **b** Estimated pdf for the last 60–30 min before birth. **c** Estimated pdf for the last 30 min before birth

An estimate of probability density function (pdf), ${\overline{\text{PDF}}}_{\text{hr}}$, for all heart rates over all episodes in each sub group was found for the last 30 min before birth, ${\overline{\text{PDF}}}_{\text{hr}}^{0}$, and the two preceding 30-min intervals, ${\overline{\text{PDF}}}_{\text{hr}}^{30}$ and ${\overline{\text{PDF}}}_{\text{hr}}^{60}$. This was done to study how ${\overline{\text{PDF}}}_{\text{hr}}$ of $\text{fhr}(n)$ changes before and after the drop in the $\text{mFHR}(t)$ observed in experiment 2. ${\overline{\text{PDF}}}_{\text{hr}}$ the last 90–60 min before birth are shown in Fig. 8a, the last 60–30 min before birth in Fig. 8b, and the final 30 min before birth in Fig. 8c.

At 90–60 min before birth, shown in Fig. 8a, all outcome groups have a peak in the 135–145 bpm region. 60–30 min before birth, a similar peak is found for the normal and NCU groups. The perinatal mortality group still has its peak at 135 bpm, although not as distinct, and the variance, $\sigma ^{2}_{\text{PDF}}$, is increased. In the last 30 min before birth, $\sigma ^{2}_{\text{PDF}}$ increases for both the normal group and the NCU group, but the peak stays within the same 135–145 bpm region. For the perinatal mortality group, $\sigma ^{2}_{\text{PDF}s_3}$, the peak has now shifted down to 110 bpm.

### Experiment 4: fetal heart rate distribution over time

To increase the visual interpretability of how the trend and spread changes over time, ${\overline{\text{PDF}}}_{\text{hr}}$ was computed using 10-min non-overlapping intervals for the last 150 min before birth, plotted together in a 3D surface plot.

**Fig. 9:**
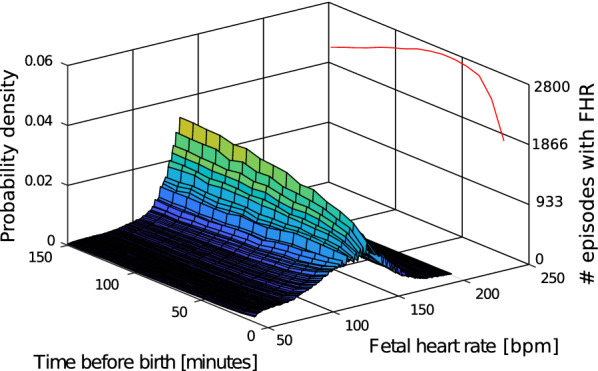
Estimated pdf changes over time, neonates defined as normal 24 h after birth

**Fig. 10:**
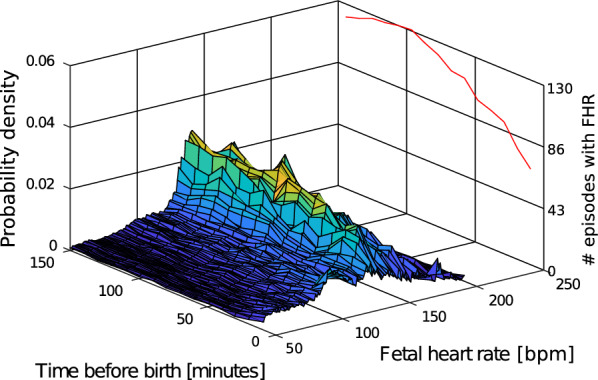
Estimated pdf changes over time, neonates admitted to NCU 24 h after birth

${\overline{\text{PDF}}}_{\text{hr}}$ over time for $s_1$ is shown in Fig. 9, and for $s_2$ in Fig. 10. The red line indicate the number of episodes containing any measured FHR signal in the corresponding time interval. For $s_1$, Fig. 9, and $s_2$, Fig. 10, the variance increase closer to birth. The number of episodes contributing the analysis is, however, lower for the NCU group than the normal group. For the perinatal mortality group, it is even smaller, and we have not included the pdf.

## Discussion

All subgroups in the dataset contain a relatively high percentage of missing data. A mean of approximately 30% missing data is seen in the normal, NCU and VEND groups, while episodes in the FSB group has a mean of 40% missing data points. The spread is, however, large, with a standard deviation of approximately 20% for all groups. FhrClean [15] removes 1–2% of the samples as they are not likely to be true FHR measurements, further increasing the amount of missing data. This removal is, however, desired as these data points are considered as noise or MHR. The missing data points may introduce an uncertainty in the analyses, but the impact is expected to be relatively small due to the large number of labours included in the study. While methods for estimation of missing data in FHR signals exists, we have, in this work, chosen to only study the measured heart rate itself as estimated signals could introduce new unwanted artefacts or skew the distribution.

The MHR/FHR ambiguity found at time points where both measurements are available stays within the same ambiguity rate as observed by Reinhard et. al. [16] for Doppler-based CTG. This indicates that the Moyo Fetal Heart Rate monitor does not wrongly register the MHR instead of the FHR more often than Doppler-based CTG, objective 1 in Table 2.

Understanding how the FHR develops differently for the different neonatal outcome groups might provide valuable insights in understanding the risk of birth asphyxia at an early phase in the labour. The FHR development the last 150 min before birth, shown in Fig. 5, shows a decrease in the measured FHR the last 150 min before birth, we call this result 2 subfinding a. As seen in Fig. 7, labours in the normal and the NCU groups follow the same trend, where a small decrease can be seen from 40 min before birth and then a larger decrease the last 10–20 min before birth. For labours in the perinatal mortality group, $s_3$, the onset of a larger decrease occurs already at 40 min before birth. The observed drop in FHR close to birth for all labours indicate that a drop is expected and should alone not lead to a premature intervention. However, the drop seems to occur earlier and more severely in newborns with a need of resuscitation. More research on new data should follow to see if it is possible to predict the risk of asphyxia based on a combination of the FHR development and the phase of the birth. The observed drop in the normal and NCU group is likely to be caused by an increased frequency and intensity of the uterine contractions as the labour progresses. The drop is larger and occur longer time before birth in the perinatal mortality group, objective 2 subfinding b, may also be caused by the increased frequency and intensity of the uterine contractions, and may indicate that the fetus is unable to cope with this increased intensity.

By studying the difference in ${\overline{\text{PDF}}}_{\text{hr}}$ in 30-min intervals during the last 90 min before birth, we observe that the variance increase for all subgroups as the labour progresses towards birth, we call this objective 3 subfinding a. The perinatal mortality group has also a larger increase in the variance than the other two groups, and the shift in peak down to 110 bpm in the last 30 min is also larger than for the other subgroups, we call this objective 3 subfinding b. As ${\overline{\text{PDF}}}_{\text{hr}}^{30}$ for the perinatal mortality group in Fig. 8a resembles the normal and NCU in Fig. 8c, lives could potentially been saved if interventions had been conducted at an earlier point in time. An overview of the results are summarised in Table 2.

By introducing the same 10-min intervals from the FHR development experiment to the computation of the ${\overline{\text{PDF}}}_{\text{hr}}$ of the heart rate, we can see that the variance gradually increases, Fig. 9. The probability for heart rates below 100 bpm also increases during the last 10-min interval before birth. A similar trend is seen for labours where the neonate is still admitted to neonatal care unit 24 h after birth, Fig. 10. The jagged shape of the pdf in Fig. 10 may be a result of too few data points. A similar illustration for the perinatal mortality group is challenging due to the low amount of episodes with this outcome.

A limitation in this study is the low number of neonates in the perinatal mortality group. In addition, not all recordings include data points all the way up until the time of birth, further reducing the amount of episodes included in the study, where the last minutes before birth is very important. The reduction in number of episodes with data close to birth can be seen on the red line to the right in Figs. 9 and 10. To increase the amount of data, a longer data collection period would be desired.

A second limitation of this work is that the internal clock in the Moyo monitor has shown to be drifting. If not calibrated often enough this can result in inaccuracies of up to 30 min in the logged time stamp. The result of this is that the heart rate presented 0–1 min before birth may in worst-case be recorded 30 min before birth for some episodes. In cases with drift in the opposite direction, a FHR may be present in the signal after the defined time of birth. Episodes with a measured FHR after the time of birth are corrected by adjusting the time of birth to the time of the last found FHR.

**Table 2:** Overview of the main findings

Objective	Subfinding	Result
1		MHR/FHR ambiguity stays within the same region as observed on Doppler-based CTG
2	a	A reduction in $\text{mFHR}(t)$ is observed the last 150 min before birth
	b	The reduction in $\text{mFHR}(t)$ for the perinatal mortality group is larger, and occurs longer time before birth
3	a	The variance of ${\overline{\text{PDF}}}_{\text{hr}}$ increases for all subgroups closer to birth
	b	A larger increase in the variance as well as a shift in the peak is observed for the perinatal mortality group

## Conclusion

In this work, we have shown that the amount of MHR/FHR ambiguity using the Laerdal Moyo fetal heart rate monitor in timepoints where both measurements are available is similar to the ambiguity previously reported in Doppler-based CTG, objective 1.

The heart rate of newborns have previously been reported to increase shortly after birth, and a corresponding drop in the measured FHR close to the time of birth is for the first time observed and reported in this work, objective 2. The observed drop in FHR the last 150 min before birth is found to be statistically significant ($p<0.05$) for labours with a normal outcome as well as labours where the newborn is still under observation 24 h after birth ($p<0.05$). A larger increase in variance and shift in peak is seen for labours in the perinatal mortality group, objective 3. Additional data are required to conclude if drop in FHR for labours ending in fresh stillbirth and early neonatal death is significant, as well as describing the differences between the groups.

We are currently working to expand the data collection to further validate these results. In future work, we will validate the findings on new datasets, and we will study the relationships between the groups in more detail. We also plan to combine the new knowledge on FHR development with automated FHR interpretation tools using signal processing and machine learning for risk predict during labour.

## Methods

### Data material

The data were collected at three hospitals in Tanzania between October 2015 and June 2018. Haydom Lutheran Hospital is located in a rural part of the country, while the Muhimbili National Hospital and Temeke Referral Hospital are located in Dar es Salaam. During the study period, data from 3711 labours were collected. At 24 h after birth, 3490 neonates were defined as normal, 185 were still admitted to neonatal care unit, 18 died within the first 24 h, and 12 were classified as fresh stillborn. 6 labours were not associated with any of the four outcomes above, and were, therefore, excluded from this work. A brief overview of some of the clinical features describing the labours, such as gestation age, maternal age, and birth weight are shown in Table 3.

**Table 3:** Overview of the dataset

Total number of labours	3711
Gender	1978 male, 1733 female
Birth weight	3275 [2950, 3500]
Maternal age	25 [21, 30]
Gestational age	39 [38, 40]
Mode of delivery	2862 SVG, 716 C/S, 20 ABD, 113 Vacuum

SVG indicates a vaginal birth, C/S a cesarean section, and ADB vagial breech. Numbers in brackets indicate first and third quartile

**Fig. 11:**
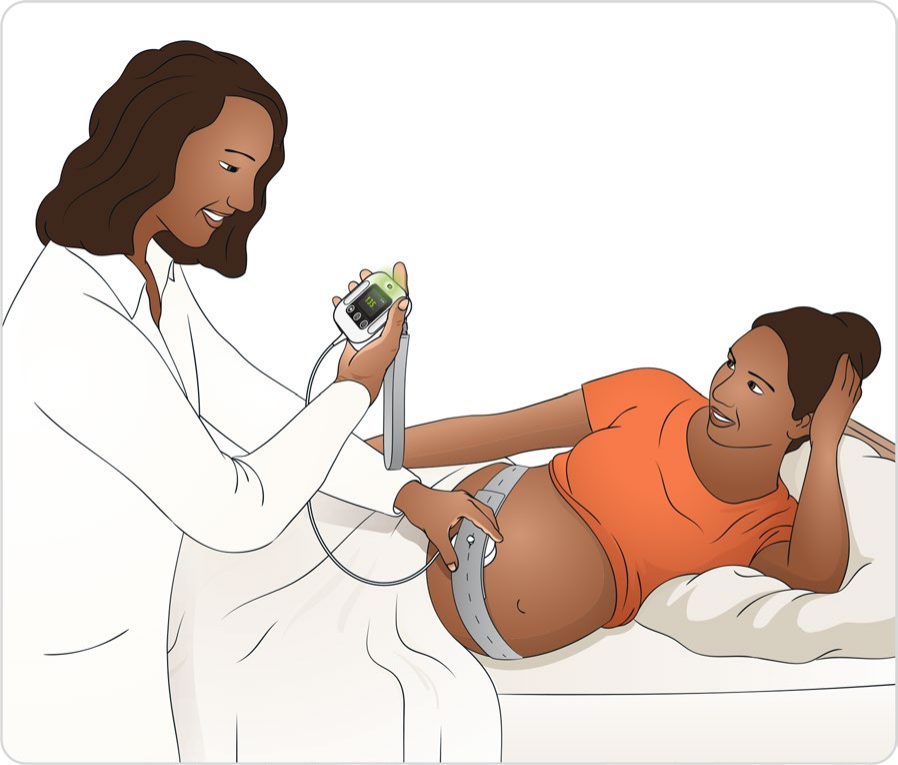
Moyo fetal heart rate monitor, Laerdal Global Health AS, Norway. Illustration reproduced with permission [17]

As the use of CTG is not feasible in LMIC settings and the availability of low-cost continuous FHR monitors are limited, the data collection was performed using the Moyo Fetal Heart Rate Monitor, illustrated in Fig. 11. The monitor is developed by Laerdal Global Health^3^, a collaborator in the Safer Birth project, especially for use in LMIC settings.

#### Moyo fetal heart rate monitor

The Moyo fetal heart rate monitor measures FHR using a 9-crystal pulsed wave Doppler ultrasound sensor operating at a frequency of 1 MHz and an intensity of less than 5 mW/$\hbox {cm}^2$. The heart rate is computed and logged twice per second, equivalent of a sampling rate of 2 Hz. In addition, the device is equipped with an accelerometer, sampled at 50 Hz, a temperature sensor and an dry-electrode ECG sensor for measurement of the maternal heart rate (MHR). The ECG sensor, used to measure the MHR requires the mother to keep one finger from each hand on the monitor. It is, therefore, suitable to intermittently assess the MHR, or to determine if the Doppler measurement captures the true FHR or if it falsely detects the MHR.

The Moyo FHR monitor is similar to Doppler-based CTG for measurement of the FHR, but it lacks a sensor to detect uterine activity. To overcome this, an approach of using the accelerometer measurements from Moyo to estimate the uterine contractions has been proposed by our research group [15]. While measurements of the MHR is typically done using a separate device in high-resource settings, the inclusion of the ECG sensor is an advantage in LMIC settings as the availability of other devices may be limited. The small size of the Moyo also allows the mother to move more freely around while the device is attached. An overview of the differences and similarities between Moyo and Doppler-based CTG can be seen in Table 4.

**Fig. 12:**
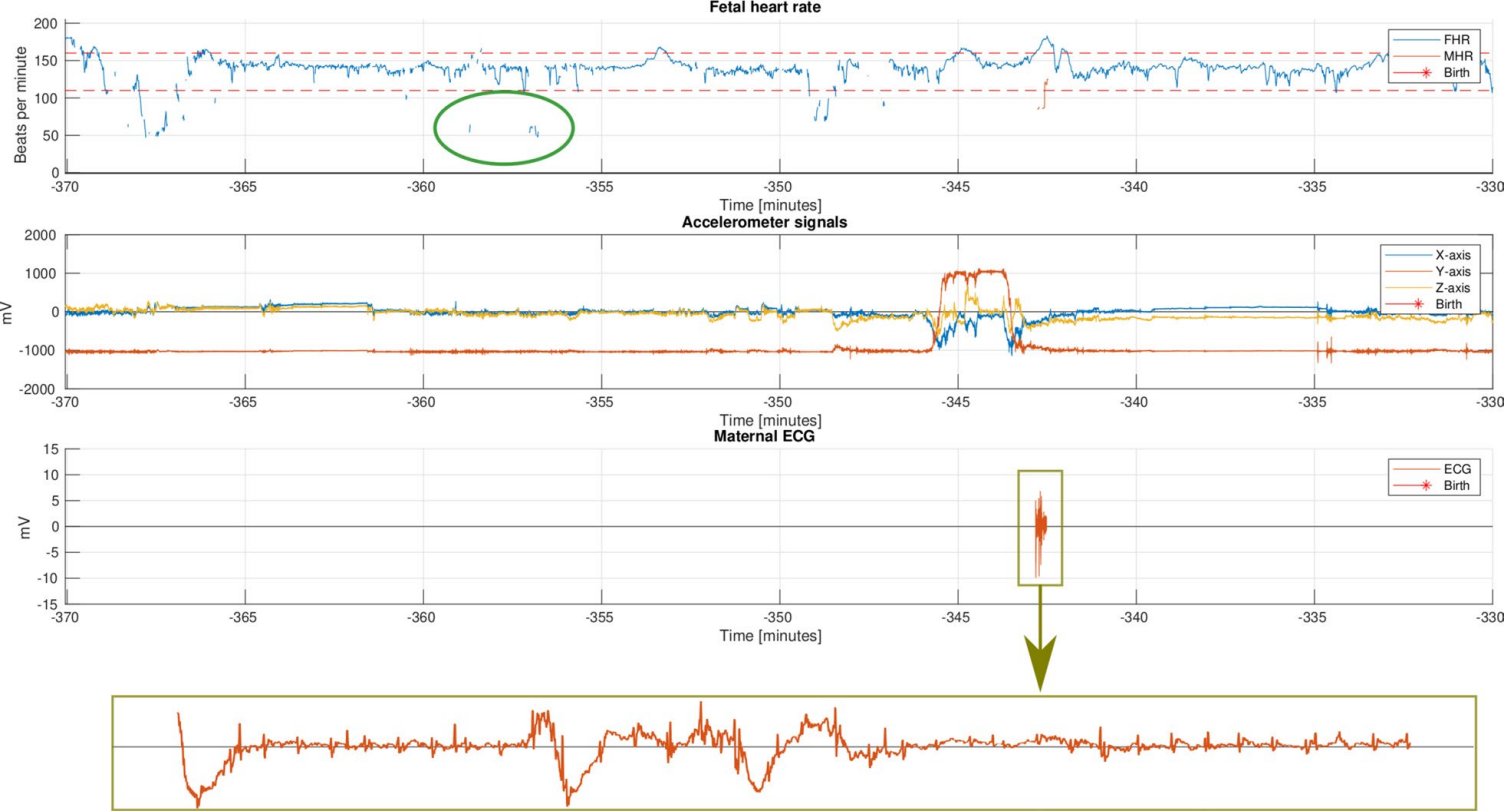
Example signals from the Moyo fetal heart rate monitor. The top plot includes the FHR, shown in blue, and MHR, shown in red. The red dashed lines indicate the normal FHR region during labour. An example of the noise removed by FhrClean is illustrated in the green circle. The second plot shows the three axes measured from the accelerometer. The MHR is intermittent measured at −343 min. A zoomed version of the maternal ECG, with visible R-waves is shown on the bottom

A segment from the signals collected using Moyo during a labour example episode is shown in Fig. 12. The top plot shows FHR and MHR, in blue and red, in relation to the time of birth. The normal region, for FHR, of $110-160$ bpm is indicated by red dashed lines. The second subplot shows movement of the sensor measured by the three axes accelerometer. The MHR is computed using the ECG signal shown in the third subplot.

**Table 4:** Comparison between some of the features of Moyo Fetal Heart Rate Monitor and Phillips Avalon FM50 Doppler-based CTG

Measurements	Moyo	CTG
FHR	Doppler	Doppler
Contractions	No*	Yes
Acceleration	Yes	No
Maternal HR	Yes	Yes
Additional features	Moyo	CTG
Allows for free maternal movement when attached	Yes	No**

* Contractions can be identified using the accelerometer [15], ** The Avalon Cordless Transducer System can be connected to allow for wireless transfer from sensors to device

### Pre-processing of data

Doppler measurements of the FHR is known to be noisy [18]. To allow for a computerised analysis of the FHR, the acquired measurements should first be preprocessed to remove unwanted artefacts, such as for example interference from the MHR [18, 19], misinterpreted harmonics [20]. The use of a portable device for FHR monitoring allows for more movement, thus potentially increasing the amount of noise. In CTG, an internal transducer can be attached directly to the fetal scalp in case of poor signal quality from the Doppler sensor on the abdomen. However, this is not possible using the Moyo FHR monitor alone.

There are three distinct patterns of noise, (i) short spikes with both higher and lower values than the baseline FHR, and (ii) longer time periods deviating from the baseline FHR in a non-physical (impossible) way, and (iii) missing data points. Short spikes are relatively easy to detect, and examples can be seen the green circles in Fig. 12 as short signal segments outside of the normal region of 110–160 bpm, illustrated using the dashed red lines. Time periods with such noise in the measured FHR signal are identified using our recently proposed method for noise identification in FHR signals [15]. The noise removal method, denoted FhrClean [15], is run on the complete dataset prior to further analysis, as illustrated by the box in Fig. 1. FhrClean first utilise forward and backward replication to fill any missing data in the measured signal. Less trustworthy time periods, meaning periods where the measurement is not likely to consist of the true FHR, are found by identifying temporary drops or peaks in the signal. A more in-depth view of the used method can be found in Urdal et. al. [15].

Estimation of missing data in FHR analyses are often done using linear interpolation [21, 22], cubic spline interpolation [23] or more complex methods such as Guassian processes [24], K-SVD [25] and shift-invariant dictionaries [26]. However, in this work, we are looking at the recorded values directly, and we can easily handle gaps in the data. To avoid any introduced effect from the estimated data in the FHR development analysis, it was decided not to perform estimation of missing data, and base our analysis on only actually measured heart rate points.

### Heart rate ambiguity

Doppler-based FHR measurements are susceptible of incorrectly picking up the MHR due to sub-optimal sensor placement [27]. If the FHR is within 5 bpm of the MHR, it can be classified as an MHR/FHR ambiguity. Since the amount of MHR/FHR ambiguity in Doppler CTG is found to be $1.22\pm 1.9$% during the first stage of labour and $6.2\pm 9.0$% during the second stage or labour [16], it may cause unwanted artefacts in digital analysis of the FHR. The option of measuring MHR using Moyo is intended to be intermittent and not continuous, the amount of measured MHR varies from labour to labour. Thus, the possibility of verifying whether the measured HR from the ultrasound Doppler is maternal or fetal is, therefore, limited. The MHR can also mimic an expected FHR, making it challenging to distinguish true MHR from true FHR signals [28].

To study the MHR/FHR ambiguity on data acquired using the Moyo fetal heart rate monitor, all time points where both signals exist are studied, indicated as the *Group analysis matching FHR/MHR* box in Fig. 1. Let $h_t$ be a vector of a FHR fhr(n) sample and a MHR sample mhr(m)

1 $$\begin{aligned} h_t = [\text{fhr}(n_t), \text{mhr}(m_t)] \end{aligned}$$

sampled with different sampling rate, here represented with a sample at the corresponding time point, *t*. Let H be the set of all such matching heart rate pairs, $h_t$

2 $$\begin{aligned} H = \lbrace h_t: \text{fhr}(n_t)>0 \cap \text{mhr}(m_t)>0 \rbrace \end{aligned}$$

The MHR/FHR ambiguity, $mhr_{amb}\in \lbrace 0, 1\rbrace$, in an episode is calculated as a fraction of the time where both signals are present, defined as

3 $$\begin{aligned} \text{mhr}_{amb} = \frac{1}{N_H}\sum _{h_t \in H} \mathbf{I }(h_t) \end{aligned}$$

where $N_H$ is the number of vectors, $h_t$,i.e. the number of matching time points, in *H*, and $\mathbf{I }(h_t)$ is an indication function given by

4 $$\begin{aligned} \mathbf{I }(h_t) = {\left\{ \begin{array}{ll} 1; \quad if &{} |h_t(1) - h_t(2)| =< T_{\text{mhr}}\\ 0; \quad if &{} |h_t(1) - h_t(2)| > T_{\text{mhr}} \end{array}\right. } \end{aligned}$$

and $T_{\text{mhr}}$ is a threshold to allow some inequalities due to the different measurement techniques.

### Fetal heart rate

Labour is normally a physical strain on both the mother and the fetus. As the labour progresses, this strain may affect the physical condition of the fetus, and can potentially be observed on the measured FHR. Analysing continuous FHR measurements from a large number of labours assessed as low-risk on admission, can potentially be used to determine if differences exist in the heart rate development between neonates with normal or adverse outcomes.

As the time period where the FHR is measured vary from labour to labour, we define the sample index, *n*, in the measured FHR signal based on the measured FHR sample rate, 2 Hz, the elapsed time, *t*, and a defined start point before birth, $t_0$, such as

5 $$\begin{aligned} n = 2 (t+t_0). \end{aligned}$$

In the following sections, we describe the *group analysis over all episodes* box in Fig. 1, utilising groups of the dataset based on neonatal status 24 h after birth.

#### Fetal heart rate development

With the use of continuous FHR monitoring in a large number of labours, it is possible to determine how the heart rate develops during labour and develop trend models. This can in turn be useful to determine how new labours progress compared to the known trend models.

The measured FHR within a defined interval, $\Delta$, in an episode, *i*, from the group, $s_k$, is extracted from the start time, *t*, and throughout the duration, $t+\Delta$. Let the trend, $\text{mFHR}_{s}(p)$, be defined as the median of all measured heart rates in the interval, of all episodes in the group, *s*

6 $$\begin{aligned} \text{mFHR}{s}(p) = \text{median}\left( \left( fhr^t_{1,s}(n), \ldots , fhr^t_{L_s,s}(n)\right) \right) \qquad n \in \lbrace t, t+\Delta \rbrace , \end{aligned}$$

where $L_s$ is the number of episodes in the group *s*, and the sampling index *p* is given by

7 $$\begin{aligned} p = \frac{1}{\Delta } (t+t_0). \end{aligned}$$

To describe the spread at each interval, the 1st and 3rd quartiles, $q_1, q_3$, called $\text{HRq}_1(t)$ and $\text{HRq}_3(t)$, are computed using the concatenated vector of all FHR in the interval, $\left( fhr^t_{1,s}(n), \ldots , fhr^t_{L_k,s}(n) \right) \forall n\in \lbrace t, t+\Delta \rbrace$.

As a normal distribution cannot be assumed, the change in $\text{mFHR}(t)$ from 150 to 40 min before birth to the last 10 min before birth will be validated using the Wilcoxon’s signed-rank test. The distribution in each group will be compared using the Kruskal–Wallis statistical test.

#### Fetal heart rate distribution

To illustrate changes in ${\overline{\text{PDF}}}_{\text{hr}}$, we utilise a normalized histogram to estimate the pdf in an interval defined by the start point, *t* and end point $t+\Delta$ for all episodes in a group. When computing two or more distributions, these can be used to identify how the distribution changes over time. Let $h_i^t(l)$ be the histogram of the measured FHR in episode *i*, in the interval with start point *t* and end point $t+\Delta$:

8 $$\begin{aligned} h^t_{s}(l) = \sum _{i \in s} h_i^t(l) \quad \forall \quad l=\lbrace 50,51,\ldots ,200 \rbrace \end{aligned}$$

Where *l* indicates the histogram variable, heart rate from 50 to 200 and *s* the outcome group. The normalized histogram, ${\bar{h}}_{s}(l)$, is then given by

9 $$\begin{aligned} {\bar{h}}^t_{s}(l) = \frac{1}{N} h^t_{s}(l), \end{aligned}$$

where *N* is the total count in $h^t_{s}(l)$.

By combining multiple normalized histograms using continuous non-overlapping intervals, both the change in trend and spread of the FHR can be visualised simultaneously in a 3D surface plot. A peak in the computed histograms will result in a visible ridge in the 3D visualisation.

## Data Availability

The datasets analysed during the current study are not publicly available due to National Tanzanian regulations. Data access might be granted upon on reasonable request to the authors.

## Abbreviations

FHR: Fetal heart rate
CTG: Cardiotocography
mFHR: Median fetal heart rate
MHR: Maternal heart rate
VEND: Very early neonatal death
FSB: Fresh stillbirth
NCU: Neonatal care unit

## Footnotes

1 http://www.saferbirths.com.

2 https://laerdalglobalhealth.com/products/moyo-fetal-heart-rate-monitor/.

3 https://laerdalglobalhealth.com.
